# Rapid Determination of Soybean Protein Content by Near-Infrared Spectroscopy Coupled with Multi-Learner Ensemble Wavelength Selection

**DOI:** 10.3390/foods15101755

**Published:** 2026-05-15

**Authors:** Weida Wang, Chunqi Wang, Baocheng Zhao, Jiayi Shi, Changan Xu, Jinming Liu

**Affiliations:** 1 College of Information and Electrical Engineering, Heilongjiang Bayi Agricultural University, Daqing 163319, China; wangweida1203@163.com (W.W.); z13361558869@163.com (B.Z.); 15941292571@163.com (J.S.); 2 College of Food, Heilongjiang Bayi Agricultural University, Daqing 163319, China; wangchunqi1996@foxmail.com; 3 College of Materials and Energy, South China Agricultural University, Guangzhou 510642, China; xuchangan@scau.edu.cn

**Keywords:** soybean, protein, near-infrared spectroscopy, wavelength selection, model interpretability

## Abstract

Soybean protein content is a key indicator of nutritional value and quality grade, and its determination is important for quality evaluation and cultivar selection. To overcome the time-consuming and costly limitations of conventional chemical assays, this study proposed a multiple linear learner ensemble importance-score wavelength selection (MLLEISWS) method to identify informative wavelengths from soybean near-infrared spectra and establish a partial least squares (PLS) model. MLLEISWS was compared with competitive adaptive reweighted sampling, successive projections algorithm, and uninformative variable elimination. Shapley additive exPlanations (SHAP) were applied to the MLLEISWS algorithm to interpret the selected wavelengths. Results showed that the PLS model developed using MLLEISWS achieved the best performance. With only 29 selected wavelengths, the coefficients of determination for the training and test sets reached 0.941 and 0.933, respectively. Root mean square errors were 0.490% and 0.514%, relative root mean square errors were 1.32% and 1.37%, and residual predictive deviation was 3.863, indicating predictive accuracy and stability. SHAP analysis showed that the selected wavelengths were located in protein-related spectral regions and corresponded to overtone and combination bands information from functional groups. MLLEISWS effectively reduced variable dimensionality while maintaining model performance.

## 1. Introduction

Soybeans are one of the most important agricultural resources worldwide. As a major grain-and-oil crop and a high-quality source of plant protein, it is widely used in food processing, feed formulation, and the oil industry [1,2,3]. Soybean seeds are mainly composed of protein, lipids, and moisture. Among these components, protein is one of the most essential nutritional constituents of soybeans. It is rich in essential amino acids required for the human body and serves as the material basis for soybean products and plant-protein products [4]. Soybean protein content not only determines nutritional value and processing suitability, but also affects the processing quality of end products [5]. Therefore, rapid and accurate determination of soybean protein content is of great significance for quality grading and cultivar selection [6]. At present, laboratory-based chemical methods such as the Kjeldahl nitrogen method and the Dumas combustion method are accurate and reliable. Specifically, the Kjeldahl nitrogen method usually involves wet-chemical procedures, including digestion, distillation, and titration, which result in a relatively long analysis time and high reagent consumption. By contrast, the Dumas combustion method is faster and more automated, but it still requires specialized combustion equipment and sample handling under laboratory conditions. Thus, despite their reliability, these methods remain limited for the rapid screening of large numbers of samples [7]. Therefore, the development of rapid and nondestructive detection techniques has become an urgent practical need in this field.

Near-infrared spectroscopy (NIRS) offers the advantages of fast measurement and nondestructive analysis. When supported by a validated calibration model established using representative samples and their reference measurements, NIRS can be used for rapid prediction and screening of specific quality attributes of agricultural products [8,9]. However, NIR spectra usually arise from overlapping absorption of multiple components. Band overlap and scattering effects often introduce substantial redundant information and significant collinearity, so direct full-spectrum (FULL) modeling generally increases the difficulty of model construction [10,11,12]. Related studies usually combine feature extraction or wavelength selection to reduce variable dimensionality and alleviate collinearity [13]. Feature extraction projects the original spectra into a low-dimensional latent variable (LV) space through variable transformation, thereby reducing model complexity. However, LVs are often difficult to map directly to specific wavelengths, which weakens the physical interpretability of spectral variables [14]. In contrast, wavelength selection removes redundant wavelengths while retaining key variables related to the target component. This strategy not only compresses the variable set but also facilitates mechanistic interpretation, and has therefore been widely adopted in NIR quantitative analysis [15].

Common wavelength selection methods include competitive adaptive reweighted sampling (CARS), successive projections algorithm (SPA), and uninformative variable elimination (UVE) [16,17,18]. CARS identifies key wavelengths through competitive sampling and adaptive reweighting. SPA reduces collinearity through stepwise projection and obtains a wavelength subset with lower redundancy. UVE removes uninformative variables according to variable stability or contribution, thereby improving model robustness. These methods have been widely applied in NIRS wavelength selection to develop high-accuracy protein detection models. Du et al. combined CARS with partial least squares (PLS) for the rapid determination of crude protein content in alfalfa feed, and the coefficient of determination ($R^{2}$) of the test set reached 0.99 [19]. Wang et al. used rice samples with different grinding particle sizes and established several machine learning models based on NIRS combined with CARS, SPA, UVE, and other wavelength selection methods. They found that, for samples with a particle size of 1.0 mm, the deep extreme learning machine model built with UVE-selected wavelengths performed best, with the $R^{2}$ values of both the validation set and the external test set exceeding 0.96 [18]. Li et al. used a portable near-infrared (NIR) spectrometer combined with CARS, SPA, UVE, and other wavelength selection methods for the quantitative analysis of oat protein content, and achieved rapid nondestructive prediction of oat protein content [20].

In addition, some researchers have examined model regression coefficients to investigate the contribution strength of each wavelength variable to the response variable. The coefficient magnitude is then used as an importance score for wavelength ranking and screening, after which a certain number of highly contributive wavelengths or those exceeding a given threshold are selected as feature wavelengths to build high-performance spectral detection models [21]. Ma et al. proposed an efficient spectral wavelength selection method based on the max-relevance min-redundancy algorithm, which successfully identified variables strongly related to the target variable while minimizing redundancy among feature variables, and achieved better spectral modeling performance for maize germination rate than CARS, SPA, and UVE [22]. Ahmed et al. evaluated and screened wavelength variables according to their importance as indicated by the regression coefficients and established a simplified PLS model, which effectively reduced the interference of redundant variables while maintaining model robustness and improving predictive performance [21]. Ong et al. proposed an improved feature wavelength evaluation method based on regression coefficients. By weighting and scoring the coefficients corresponding to each wavelength in the regression model, they enhanced the discrimination of key informative spectral regions and achieved rapid determination of chlorophyll content in sugarcane leaves [23].

Although the above methods have shown good performance in dimensionality reduction and model performance improvement, soybean protein represents a multicomponent coupled system with strongly overlapped absorption peaks. Its spectral variables usually exhibit both strong correlation and redundancy. As a result, even after screening, the selected feature wavelengths may still contain overlapping information. Under such circumstances, a single feature selection method or a single coefficient-based wavelength scoring method often struggles to balance feature stability, predictive accuracy, and variable compression simultaneously. Moreover, even when a relatively good feature subset is obtained, it is still necessary to further clarify the validity of the selected spectral regions and their directional effects on prediction results from the perspectives of mechanism and contribution, so as to enhance the credibility of the results [24]. Therefore, to address the above issues, this study developed a feature wavelength selection and modeling strategy that considers prediction accuracy, variable compression, and result interpretability. First, multiple linear learners were used to evaluate the importance of wavelength variables from different perspectives, and an ensemble strategy was adopted to generate more stable integrated wavelength scores, thereby reducing the dependence on a single selection criterion. Subsequently, the FULL variables were compressed into a small number of representative feature wavelengths, and a PLS quantitative prediction model was established using the selected variables to alleviate the negative effects of information redundancy and multicollinearity on model performance. Finally, Shapley additive exPlanations (SHAP) was introduced to analyze the direction and magnitude of the contribution of key wavelengths to model predictions and to evaluate whether the selected wavelengths were chemically consistent with protein-related absorption regions, thereby improving the credibility and interpretability of the model results.

Based on the above analysis, this study aimed to (1) construct a multiple linear learner ensemble importance-score wavelength selection (MLLEISWS) method, based on five linear learners, optimize NIRS feature wavelengths for soybean protein, and compare its modeling performance with those of CARS, SPA, and UVE to verify the effectiveness of MLLEISWS; (2) apply SHAP to the soybean protein-related feature wavelengths obtained by MLLEISWS, and analyze the magnitude and direction of the contributions of key wavelengths to prediction results, thereby improving the credibility of the conclusions; and (3) establish a high-accuracy prediction model for soybean protein content that can meet the requirements of rapid detection, and provide methodological support for quality grading and breeding screening.

## 2. Materials and Methods

### 2.1. Soybean Sample Collection

**Figure 1:**
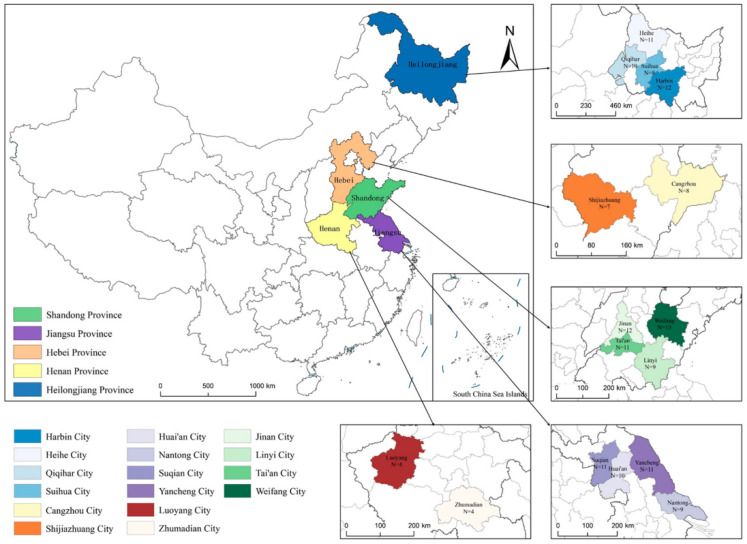
Map of Sample Origin Locations.

To enhance the geographical diversity and representativeness of the sample set, soybean samples were collected from different soybean-producing regions across China to establish a multi-origin sample set. The distribution of sampling regions and sites is shown in Figure 1. The sampling locations included Suqian, Huai’an, Yancheng, and Nantong in Jiangsu Province; Weifang, Jinan, Tai’an, and Linyi in Shandong Province; Cangzhou and Shijiazhuang in Hebei Province; Luoyang and Zhumadian in Henan Province; and Heihe, Harbin, Suihua, and Qiqihar in Heilongjiang Province. The sample number at each site is shown in Figure 1. After collection, all samples were screened in a uniform manner. Damaged, moldy, insect-infested, and obviously malformed seeds were removed, and only soybeans with intact, smooth seed coats and good appearance were retained. A total of 150 soybean samples were finally obtained. To reduce the influence of storage-condition differences on subsequent physicochemical measurements and NIRS acquisition, all samples were vacuum-sealed as intact seeds. Before analysis, the seeds were ground into powder using an FW100 grinder (Taisite, Tianjin, China) and then sieved through a 0.25 mm mesh. The resulting powder was stored in a cool, dry, and light-protected environment.

### 2.2. Spectral Acquisition and Processing

NIR diffuse reflectance spectra of soybean powder samples were acquired using a TANGO NIR spectrometer manufactured by Bruker (Bremen, Germany). Before use, the instrument was preheated for 30 min to ensure stable operation of the light source and the system. Spectra were collected in integrating sphere diffuse reflectance mode over a wavenumber range of 11,542 cm^−1^ to 3946 cm^−1^ with a resolution of 8 cm^−1^. Before measurement, a background spectrum was collected once per hour and used for background correction. During sample loading, the powder was evenly packed into the sample cup to a height of no less than one-third of the cup height, and the sample powder was then compacted using the sample cup lid. To reduce random error, each sample was scanned three times, and the average spectrum of the three scans was used as the representative spectrum for subsequent analysis.

In NIRS analysis, the acquired raw spectra (RAW) contain not only chemical information related to the target component, but also nonchemical interference introduced by instrumental response drift, environmental fluctuations, and differences in sample particle size. Such interference is often manifested as baseline drift, increased high-frequency noise, and local band distortion, which weaken the correspondence between spectral variables and the target component and reduce model prediction accuracy [25]. To suppress these effects and improve data quality, multiple preprocessing strategies were applied to the raw spectral data in this study, including Savitzky–Golay smooth (SG) [26], multivariate scattering correction (MSC) [27], standard normal variate (SNV) [28], wavelet denoising (WD) [29], Fourier transform (FT), spectral deconvolution (Deconv) and moving average smoothing (MAS). Furthermore, the differences between single preprocessing methods and cross-combined preprocessing strategies were further investigated to identify the optimal preprocessing method. All preprocessing methods were evaluated using PLS regression combined with repeated 10-fold cross-validation. This procedure was repeated 100 times. The specific parameter settings for these preprocessing methods were as follows. SG was performed using a 7-point window, a third-order polynomial, and a zero-order derivative. For MSC, each sample spectrum was linearly fitted against the reference spectrum to correct scattering effects. SNV was applied to each individual spectrum by mean-centering and scaling to unit standard deviation. WD was implemented using the coif3 wavelet with a decomposition level of 10, and the detail coefficients were denoised using the universal threshold with soft-threshold shrinkage. For FT denoising, the cutoff frequency ratio was set to 0.1, thereby retaining low-frequency spectral components while removing high-frequency noise. Deconv was performed using a kernel size of 10 and σ = 1. MAS was conducted using a moving window of 3 spectral points. In this study, the PLS model was implemented using the plsregress function in MATLAB. The number of LVs was optimized by Monte Carlo Cross-Validation (MCCV) based on the minimum mean predicted residual error sum of squares (PRESS) value. The evaluation indices included the cross-validation coefficient of determination ($R_{cv}^{2}$), the root mean square error of cross-validation (RMSECV), and the residual predictive deviation of cross-validation (RPDCV). Based on this, the optimal preprocessing scheme was obtained.

### 2.3. Determination of Protein Content

According to the Chinese national standard Determination of Protein in Foods [30], soybean protein content was determined using the Kjeldahl nitrogen method. Approximately 0.2000 ± 0.0005 g of soybean powder was weighed on nitrogen-free weighing paper and placed into a dry digestion tube. Then 0.4 g copper sulfate, 6.0 g potassium sulfate, and 10 mL concentrated sulfuric acid were added. The digestion was carried out in an HYP-320 intelligent digestion furnace (XianJian Inspection Instrument, Shanghai, China) with gradual heating until the digest became clear and transparent. The digestion tube was then removed and cooled to room temperature. After that, the nitrogen content in the digest was measured using a KDN-18K automatic Kjeldahl analyzer (XianJian Inspection Instrument, Shanghai, China). The final reference protein content used for spectral modeling was calculated as the average of three independent Kjeldahl nitrogen method determinations. For each sample, the differences among the three replicate measurements were generally less than 0.05 percentage points. The measured nitrogen content was substituted into the protein conversion formula to calculate the protein content of the sample. The protein calculation formula was as follows:

(1) $$X=\frac{(V_{1}-V_{2})\times c\times 0.0140}{m\times V_{3}/V_{4}}\times F\times 100,$$

where X is the protein content of the sample, $V_{1}$ is the volume of standard hydrochloric acid titrant consumed by the sample solution, $V_{2}$ is the volume of standard hydrochloric acid titrant consumed by the reagent blank, c is the concentration of the standard hydrochloric acid titration solution, m is the sample mass, $V_{3}$ is the volume of digest aliquot taken for analysis, $V_{4}$ is the final volume of the digestion solution, and F is the protein conversion factor, which was set to 6.25 in this study.

### 2.4. Wavelength Selection Methods

#### 2.4.1. Multiple Linear Learner Ensemble Importance-Score Wavelength Selection Method

The MLLEISWS method aims to improve the robustness of wavelength importance ranking through joint evaluation by multiple learners, thereby reducing the bias in wavelength assessment that may arise from a single model under strong collinearity, noise interference, and outlier effects. Under this mechanism, wavelengths that receive high scores only occasionally from a single learner are less likely to remain dominant in the integrated score, whereas wavelengths that consistently show high importance across multiple learners are more likely to obtain stable high rankings. The core idea of the algorithm is as follows: First, five linear learners were selected, namely forward stagewise regression (FSR) [31], Huber regression (Huber) [32], least absolute shrinkage and selection operator (LASSO) [33], PLS and ridge regression (Ridge) [34]. These learners assess the contribution of each wavelength to the response variable from different perspectives, including sparse screening, robust fitting, and collinearity suppression and so on. Among them, FSR, Huber, LASSO, and Ridge were characterized by the absolute values of regression coefficients or regression slopes, whereas PLS was characterized by the variable importance in projection (VIP). On this basis, the regression coefficients, regression slopes, and VIP values produced by the five learners were normalized, and the normalized importance scores at each wavelength were summed to obtain the integrated importance score of MLLEISWS. Based on the resulting scores, the FULL wavelengths were ranked in descending order, and candidate wavelengths were progressively introduced according to their importance from high to low. The optimal subset of feature wavelengths was ultimately determined using the minimum RMSECV of the PLS model under 10-fold cross-validation as the selection criterion.

The detailed steps of MLLEISWS are as follows:

Step 1: Five linear learners, FSR, Huber, LASSO, PLS, and Ridge, were first selected to score wavelength importance.

(1) FSR learner: A forward stagewise micro-increment update strategy was adopted. At each step, the correlation between each wavelength variable and the current residual was calculated. The variable with the largest absolute correlation was selected, and its coefficient was incrementally updated with step size *ν*. The residual was then updated simultaneously. Iteration continued until the maximum number of steps was reached or the maximum correlation dropped below a threshold. The absolute value of the final coefficient, denoted as $\left|\beta_{FSR}\right|$, was used as the wavelength importance.

(2) Huber learner: Univariate Huber robust regression was conducted for each wavelength separately. Specifically, a regression model $y∼x_{j}$ was fitted for each wavelength, and the absolute regression coefficient, $\left|\beta_{Huber}\right|$, was taken as the importance score of that wavelength.

(3) LASSO learner: $L_{1}$—regularized sparse regression was performed, and the optimal regularization parameter λ was selected by 10-fold cross-validation. The sparse regression coefficients thus obtained were used to evaluate wavelength importance, which was expressed as the absolute value of the regression coefficient $\left|\beta_{LASSO}\right|$.

(4) Ridge learner: $L_{2}$—regularized regression was employed, and the optimal regularization parameter λ was determined by 10-fold cross-validation. The model was then fitted on the full training set to obtain the regression coefficients, and $\left|\beta_{Ridge}\right|$ was used to represent wavelength importance.

(5) PLS learner: PLS modeling was performed, and the optimal number of latent variables was determined by 10-fold cross-validation. The PLS model was then refitted on the full training set, and the VIP was calculated according to the weights of each wavelength on the latent variables and the explanatory contribution of each latent variable to the response variable. $VIP_{PLS}$ was used as the measure of wavelength importance.

Step 2: Owing to differences in the numerical range and scale of the raw importance indicators produced by different learners, normalization was first performed on the raw importance indicator of each learner at each wavelength to ensure fusion compatibility. These indicators were then mapped to the interval [0, 1], thereby generating the wavelength importance scores corresponding to each learner [35]. The calculation formula was as follows:

(2) $$s_{k}(\lambda_{j})=\frac{I_{k}(\lambda_{j})-\min(I_{k})}{\max(I_{k})-\min(I_{k})},\;k\;=\;1,\;\ldots ,\;5,$$

where $s_{k}(\lambda_{j})$ denotes the normalized wavelength importance score of the *k*-th learner at the *j*-th wavelength, $\lambda_{j}$. $I_{k}(\lambda_{j})$ denotes the corresponding raw importance value of the *k*-th learner at wavelength $\lambda_{j}$. $\max(I_{k})$ and $\min(I_{k})$ denote the maximum and minimum raw importance values of the *k*-th learner over the full wavelength range, respectively. For the FSR, LASSO, and Ridge learners $I_{k}(\lambda_{j})$ is defined as the absolute value of the corresponding regression coefficient. For the Huber learner, $I_{k}(\lambda_{j})$ is defined as the absolute value of the regression slope. And for the PLS learner, $I_{k}(\lambda_{j})$ is defined as the VIP value. *K* represents the number of learners involved in wavelength importance scoring, and in this study, *k* = 5.

On this basis, the wavelength importance scores from the five linear learners were summed to obtain the MLLEISWS importance score. The formula was as follows:

(3) $$IES(\lambda_{j})=\sum_{k=1}^{K}s_{k}(\lambda_{j}),$$

where $IES(\lambda_{j})$ denotes the integrated ensemble score of the *j*-th wavelength, $s_{k}(\lambda_{j})$ is the normalized wavelength-importance score obtained from the *k*-th learner.

**Figure 2:**
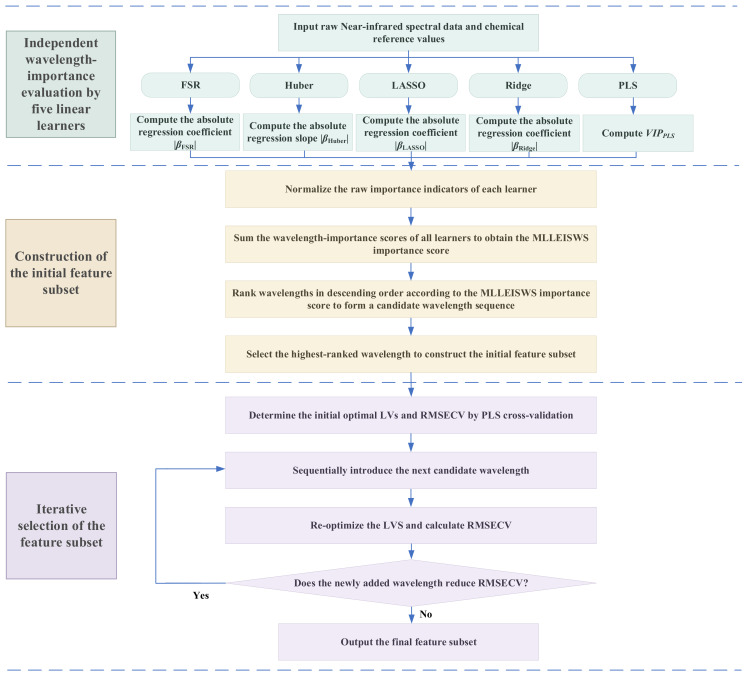
Schematic flowchart of the MLLEISWS algorithm.

Step 3: All wavelengths in the FULL were ranked in descending order according to the MLLEISWS score to form a candidate wavelength sequence. The wavelength with the highest score was first selected as the initial wavelength set. Then, the optimal number of components in the PLS model was searched by 10-fold cross-validation, and RMSECV was used as the evaluation criterion for the current model. Based on the ranking of the candidate wavelength sequence, the subsequent wavelengths were introduced into the current wavelength set one by one from the highest to the lowest score. Each time a new wavelength was added, a new PLS model was established, and the corresponding RMSECV was calculated. If the newly added wavelength led to a further decrease in RMSECV, it was retained, and the current optimal feature subset was updated accordingly. If RMSECV failed to decrease for the first time, the subsequent introduction of wavelengths was terminated. Finally, the wavelength subset corresponding to the turning point immediately before RMSECV changed from continuous decrease to no further decrease was defined as the optimal feature wavelength set selected by the MLLEISWS algorithm. The algorithm flow is shown in Figure 2.

#### 2.4.2. Classical Wavelength Selection Methods

CARS, SPA, and UVE are three classical feature wavelength selection methods widely used in NIRS analysis. They achieve variable compression from the perspectives of competitive screening, decollinearity, and removal of uninformative variables, respectively. CARS is based on Darwin’s principle of survival of the fittest. Using PLS regression coefficients as the basis, it generates candidate variable subsets through Monte Carlo iterative sampling and gradually eliminates variables with small absolute regression coefficients by an exponentially decreasing function. Adaptive reweighted sampling is then used to reinforce the retention of key variables. Finally, the subset with the minimum RMSECV is selected as the optimal wavelength set [16]. In this study, the CARS algorithm was implemented with 50 Monte Carlo sampling runs, and the maximum number of PLS LVs was set to 20. The candidate wavelength subsets generated during the CARS procedure were evaluated using 10-fold cross-validation, and the subset with the lowest RMSECV was selected as the optimal CARS wavelength subset.

SPA reduces multicollinearity among variables through iterative projection. Starting from an initial wavelength, the algorithm progressively computes the projection residuals of candidate variables relative to the selected variable set, and preferentially incorporates wavelengths with larger projection residuals into the set, thereby obtaining a candidate wavelength subset with lower redundancy. Subsequently, a multiple linear regression model is constructed, and the PRESS is obtained by cross-validation. RMSECV is calculated accordingly as the evaluation criterion, and the wavelength subset with the minimum error is selected as the optimal subset [36]. In this study, the parameter settings of the SPA were as follows. For SPA, autoscaling was applied before variable selection. The number of selected wavelengths was searched within the allowable range, smaller than the number of training samples. The final wavelength subset was determined according to the minimum RMSECV obtained from cross-validation on the training set.

UVE is a variable selection method based on PLS. It appends random noise variables to the original spectral matrix and constructs a PLS model to compare the stability of the regression coefficients of the original variables with that of the noise variables. Wavelengths with low stability are considered uninformative and are therefore eliminated, which reduces model complexity and improves modeling accuracy [17]. In this study, the parameter settings of the UVE algorithm were as follows. A total of 150 Monte Carlo sampling iterations was performed, and the number of artificial noise variables was set equal to the number of original spectral variables. The stability of each variable was evaluated based on the PLS regression coefficients obtained during repeated modeling, with the artificial noise variables used as a reference for identifying uninformative variables. The final wavelength subset was determined according to the minimum RMSECV obtained by cross-validation on the training set.

### 2.5. Model Interpretability

SHAP is a game-theory-based model interpretation method used to quantify the contribution of each input feature to the model prediction. Its theoretical basis is the idea of Shapley values. Across different feature subsets, the change in model output resulting from the inclusion of a given feature is evaluated, and the marginal contributions over all possible subsets are weighted and averaged to obtain the contribution and importance of that feature [37]. The calculation formula is as follows:

(4) $$\varphi_{i}=\sum_{S\subseteq N\backslash \left\{i\right\}}\frac{\left|S\right|!(\left|N\right|-\left|S\right|-1)!}{\left|N\right|!}\left[f(S\cup \left\{i\right\})-f(S)\right],$$

where $\varphi_{i}$ denotes the Shapley value of feature i, S denotes a feature subset, N denotes the complete feature set, and f(S) denotes the model prediction based on feature subset S. By calculating the marginal contribution of feature i before and after its inclusion under different subsets S, and then taking a weighted average of the marginal contributions over all possible subsets S, $\varphi_{i}$ is obtained. In this study, SHAP was used to interpret the output of the soybean protein prediction model and quantify the contribution of input features to prediction results, thereby enabling a deeper understanding of the basis of model decisions, revealing global feature importance, and improving model credibility.

### 2.6. Modeling Methods and Evaluation

To establish a quantitative relationship between NIRS spectra and soybean protein content, PLS regression was adopted in this study. PLS projects the original spectral variables into LV space and establishes a regression model with physicochemical indices in that space. It can effectively alleviate collinearity while reducing dimensionality, making it suitable for high-dimensional and collinear NIRS data [38]. The number of LVs determines model complexity and predictive ability. Too many LVs may lead to overfitting, whereas too few may result in insufficient information extraction [39]. Therefore, MCCV was used in this study, and PRESS was taken as the criterion for selecting the number of LVs. The number of LVs corresponding to the minimum PRESS was selected as the optimal setting.

Model performance was comprehensively evaluated using $R^{2}$, root mean square error (RMSE), relative root mean square error (rRMSE), and residual predictive deviation (RPD). $R^{2}$ measures the degree to which the model explains the variation in the response variable. RMSE measures prediction error. rRMSE is the ratio of RMSE to the mean value. RPD evaluates model reliability and discrimination ability. The formulas for $R^{2}$, RMSE, rRMSE, and RPD are as follows:

(5) $$R^{2}=1-\frac{\sum_{i=1}^{n}{\left(y_{i}-{\hat{y}}_{i}\right)}^{2}}{\sum_{i=1}^{n}{\left(y_{i}-\bar{y}\right)}^{2}},$$

(6) $$RMSE=\sqrt{\frac{1}{n}\sum_{i=1}^{n}{\left(y_{i}-{\hat{y}}_{i}\right)}^{2}},$$

(7) $$rRMSE=\sqrt{\sum_{i=1}^{n}{\left(y_{i}-{\hat{y}}_{i}\right)}^{2}/n\bar{y}},$$

(8) $$RPD=\sqrt{\sum_{i=1}^{n}{\left(y_{i}-\bar{y}\right)}^{2}/\sum_{i=1}^{n}{\left(y_{i}-{\hat{y}}_{i}\right)}^{2}},$$

where $y_{i}$ denotes the measured reference value of the *i*-th sample, ${\hat{y}}_{i}$ denotes the corresponding predicted value, $\bar{y}$ denotes the mean value of the measured reference values in the corresponding dataset, and n represents the number of samples used for model evaluation.

### 2.7. Software and Computational Environment

MATLAB R2023b (MathWorks, Natick, MA, USA) was used for spectral preprocessing, wavelength selection, MCCV-based outlier identification, PLS modeling, statistical analysis, and figure generation. All computations were performed under Microsoft Windows 11.

## 3. Results and Analysis

### 3.1. Data Analysis and Processing

#### 3.1.1. Analysis of Protein Content Data

**Figure 3:**
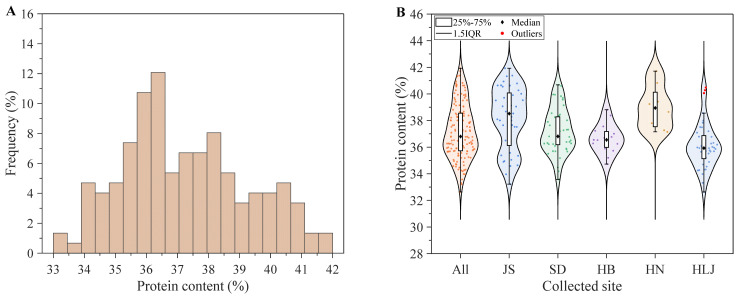
Histogram (**A**) and violin plot (**B**) of soybean protein content. JS, SD, HB, HN, and HLJ represent Jiangsu, Shandong, Hebei, Henan, and Heilongjiang, respectively. All denotes all samples.

The soybean samples overall showed an approximately normal distribution of the protein content. As shown in Figure 3A, with a peak around 35.5–36.5% and an overall range of approximately 33–42%. The violin plot in Figure 3B further shows that soybean samples from different provinces differed not only in overall protein level, but also in their internal density distributions. From the overall distribution, protein content was mainly concentrated in the range of 35–38%, indicating a relatively dense distribution of samples in this interval. At the same time, a certain number of samples were also distributed in the ranges of 33–35% and 38–42%, suggesting a relatively wide overall coverage.

Samples from Jiangsu and Henan showed relatively high overall protein levels. The Jiangsu group exhibited a wide distribution range, with a certain density in both the lower and higher value regions, indicating strong within-province variability. The Henan group was mainly concentrated on the higher protein range and showed a more compact distribution, indicating relatively low within-group dispersion. Samples from Shandong were at an intermediate level, mainly distributed between 36% and 38%, with moderate dispersion. In contrast, samples from Hebei and Heilongjiang had relatively lower medians. The violin plot for Hebei was relatively narrow, indicating a concentrated distribution and low within-group fluctuation. The Heilongjiang group was mainly concentrated around 35–37%, but a small number of high-value outliers were also observed, indicating that the overall protein content of this group was relatively low while still including a few high-protein samples.

Overall, the protein content distribution was relatively concentrated with a reasonable gradient range, providing sufficient coverage for subsequent model construction. More specifically, these regional differences may be related to ecological conditions, breeding orientation, and processing demand in different production areas. The northeastern production region, represented by Heilongjiang, mainly includes high-oil or dual-purpose protein-and-oil materials. The Huang-Huai-Hai soybean region, represented by Hebei, Shandong, and Henan, covers multiple uses including food, feed, and oil processing. Among them, the Henan samples showed relatively high protein content, which may be related to local demand for food processing applications or high-protein breeding. The lower Yangtze River region, represented by Jiangsu, has a relatively high proportion of high-protein food-grade soybeans. Therefore, this study adopted a multi-ecological-zone and multi-origin sampling design to expand the variation range of protein content and enhance sample representativeness, thus providing a more adequate data basis for subsequent NIR quantitative model development.

#### 3.1.2. Spectral Preprocessing

The results of different preprocessing methods are shown in Table 1. WD achieved the best overall preprocessing performance for this dataset, with an $R_{cv}^{2}$ of 0.901, an RMSECV of 0.582%, and an RPDCV of 3.613. In contrast, MSC, SNV, and their related combinations showed noticeably lower $R_{cv}^{2}$ values and higher RMSECV values, indicating that scatter-correction-based preprocessing did not improve model performance for this dataset. Preprocessing methods mainly aimed at smoothing or signal enhancement, such as SG, WD, FT, Deconv, and MAS, all showed some improvement over the RAW. Taken together, these results suggest that the major spectral interference in this study was more likely to come from random noise or local band perturbations. WD suppresses high-frequency detail components through multiscale decomposition and thresholding, thereby reducing random noise while preserving the band shapes and subtle spectral structures of absorption bands. This is favorable for LV extraction and improves predictive performance [40]. Therefore, WD was selected as the preprocessing method for subsequent modeling.

**Figure 4:**
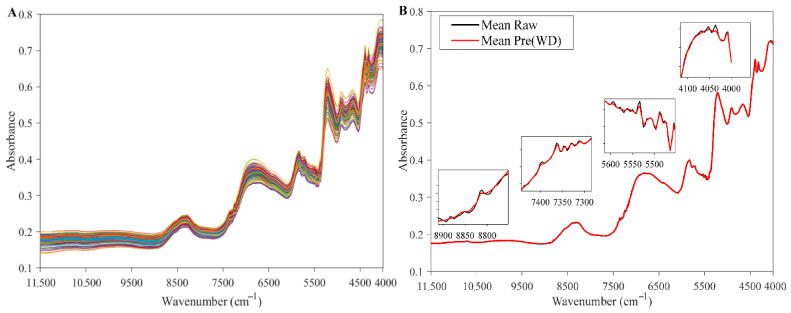
WD-preprocessed spectral data (**A**) and comparison of the mean spectra before and after preprocessing (**B**).

Figure 4A shows the spectra after WD preprocessing. The overall spectral trends of all samples were consistent, and the curves exhibited good smoothness across the FULL range. Figure 4B further illustrates the effect of WD from the perspective of mean spectra. The black line represents the mean RAW, and the red line represents the mean WD-preprocessed spectrum. The two curves were highly consistent in the peak positions of the main absorption bands, peak-shape characteristics, and the overall spectral trend, indicating that the WD process did not cause any obvious change in the macroscopic band structure. The local enlarged view in Figure 4B further reveals the effect of WD on spectral details. The small jagged fluctuations and high-frequency oscillations visible in the RAW were clearly reduced after WD treatment, and spectral continuity and smoothness were improved. Meanwhile, the main absorption peaks and shoulder peaks features remained essentially unchanged. These results indicate that WD mainly suppresses random noise and local perturbations while preserving the low-frequency band structure that reflects chemical absorption information [41].

From a modeling perspective, this means that WD can improve the signal-to-noise ratio of the spectra while minimizing the loss of useful absorption information and reducing the adverse effects of noise-induced redundant fluctuations and variable collinearity on modeling. On the one hand, after noise reduction, PLS can extract LVs related to the target component more easily. On the other hand, the reduction in irregular local fluctuations helps alleviate overfitting to noise, thereby improving model robustness and generalization ability. Considering both the cross-validation indices in Table 1 and the spectral characteristics shown in Figure 4, WD showed superior preprocessing performance for the present dataset and was therefore selected for subsequent modeling.

#### 3.1.3. Outlier Removal

**Figure 5:**
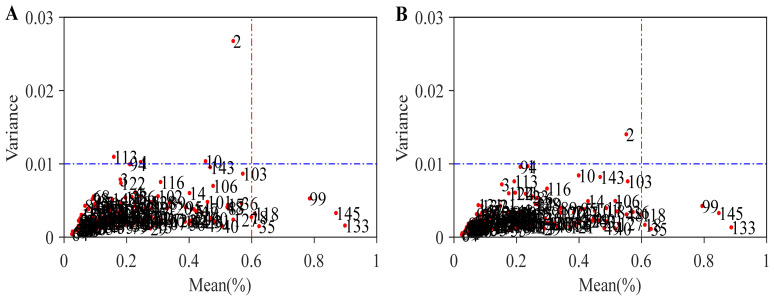
Residual mean–variance distribution calculated from the raw spectra (**A**) and preprocessed spectra (**B**).

Outlier samples are often accompanied by large prediction residuals, which can markedly reduce the fitting and generalization ability of the calibration model. Spectral preprocessing alone is usually insufficient to eliminate such errors completely. Therefore, a residual mean-variance distribution method based on MCCV was used in this study to identify outlier samples. This method identifies outliers from both spectral information and physicochemical response, thereby reducing the risk of missed detection [42]. For MCCV-based outlier detection, all candidate samples were used for preliminary sample-quality screening. PLS was adopted as the base model, and the optimal number of LVs was selected within the range of 5–20 according to the PRESS criterion. During the MCCV procedure, 1000 Monte Carlo iterations were performed. The absolute prediction residuals of each sample were recorded across repeated iterations. After all iterations, the mean and variance of the residuals were calculated for each sample to characterize its persistent prediction deviation and prediction instability. In this study, samples with a residual mean deviation greater than 0.6 or a residual variance deviation greater than 0.01 were defined as outliers and removed. These two thresholds were not used as universal fixed criteria, but were empirically determined according to the residual mean-variance distribution characteristics of the present dataset. Specifically, they were used to identify samples that clearly deviated from the main sample population while preserving the representativeness of the entire sample set. The results showed that 10 outlier samples were identified and removed under RAW conditions, as shown in Figure 5A. After WD preprocessing, the number of outliers decreased to 7, as shown in Figure 5B. At the same time, the mean-variance distribution of sample residuals after preprocessing became more concentrated overall, and the deviation of some samples was reduced, which further demonstrates the effectiveness of WD preprocessing. In particular, samples 1, 10, and 113 were effectively corrected after WD preprocessing. It is worth noting that sample 2 exhibited high residual variance both before and after WD preprocessing, indicating a relatively prominent abnormality. Although the deviation of this sample decreased after WD preprocessing, it still remained outside the outlier threshold. This suggests that WD can alleviate abnormal fluctuations to some extent, but it is difficult to completely eliminate the influence of strongly abnormal samples.

#### 3.1.4. Sample Set Partitioning

In this study, spectral preprocessing and outlier identification were used as preliminary data quality screening steps before model construction to reduce spectral noise, sample preparation effects, and abnormal reference values. After screening, the retained 143 samples were randomly divided into training and test sets at a 4:1 ratio for subsequent modeling and evaluation. The training set contained 115 samples and was used for model construction, while the test set contained 28 samples and was used for independent validation. The partitioning results are shown in Table 2. For all samples, the protein content ranged from 32.63–41.93%, with a mean of 37.233%, a standard deviation of 2.027%, and a coefficient of variation of 5.40%. For the training set, the protein content ranged from 32.63–41.93%, with a mean of 37.161%, a standard deviation of 2.031%, and a coefficient of variation of 5.50%. For the test set, the protein content ranged from 33.96% to 41.33%, with a mean of 37.527%, a standard deviation of 2.022%, and a coefficient of variation of 5.40%. The value ranges of the two subsets were generally consistent with those of the full dataset, and the training set provided good coverage of the concentration gradient represented in the test set.

**Figure 6:**
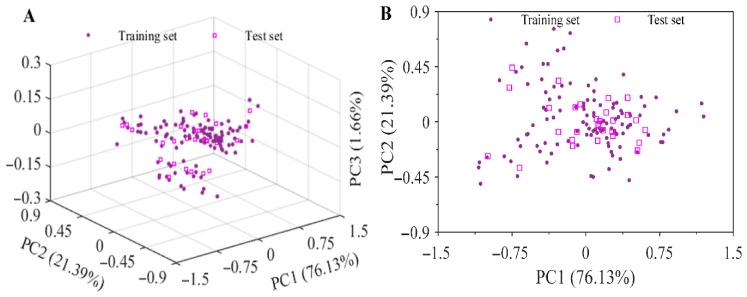
Distribution of principal components in three-dimensional (**A**) and two-dimensional (**B**) space. PC1, PC2, and PC3 represent the first, second, and third principal components, respectively.

Principal component analysis was then performed on the preprocessed spectra of the training and test sets, as shown in Figure 6. The contribution rates of PC1, PC2, and PC3 were 76.13%, 21.39%, and 1.66%, respectively, with a cumulative contribution rate of 99.18%. The training and test samples basically overlapped in principal component space, with no obvious separation observed, indicating that the two subsets showed relatively consistent coverage of the spectral feature space. Overall, the two subsets showed only small differences in mean protein level, dispersion, and variability, indicating that this partitioning strategy had good representativeness in terms of both concentration gradient and spectral features, and could provide a reliable data basis for establishing a robust protein content prediction model.

### 3.2. Feature Wavelength Selection

**Figure 7:**
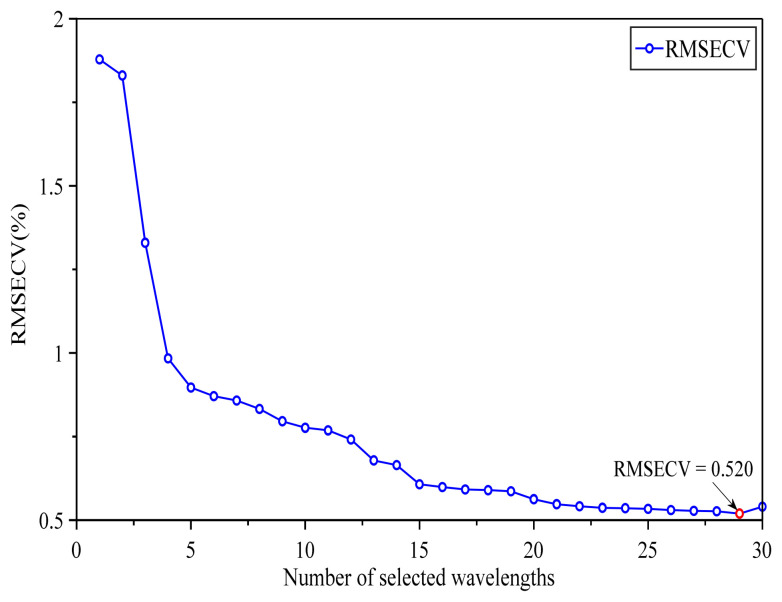
Variation in RMSECV with the Number of Selected Feature Wavelengths during the MLLEISWS Process.

**Figure 8:**
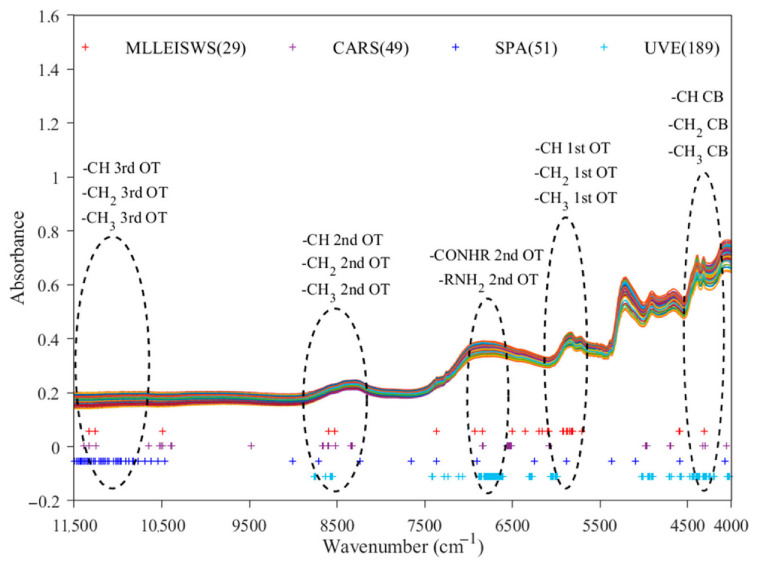
Distribution of feature wavelengths selected by different wavelength selection methods.

To extract effective spectral variables related to soybean protein content, feature screening was first conducted using MLLEISWS. In this method, candidate variables were introduced sequentially in descending order based on the wavelength importance scores generated by the MLLEISWS method, and the optimal feature wavelength subset was determined by comparing the RMSECV values corresponding to different wavelength variable subsets. To further illustrate how MLLEISWS determined the optimal feature subset, the relationship between the number of selected wavelengths and RMSECV was plotted, as shown in Figure 7. The results showed that, as highly important wavelengths were progressively incorporated into the model, RMSECV exhibited an overall decreasing trend, indicating that the introduced variables continuously enhanced the cross-validation performance of the model. When the number of selected wavelengths increased to 29, RMSECV reached its minimum value of 0.520. However, after the 30th wavelength was added, RMSECV increased instead, indicating that the new variable did not further improve model performance and may have introduced redundant information or noise. Therefore, the subset containing 29 wavelength variables was defined as the optimal feature wavelength set selected by MLLEISWS. Compared with the 1845 original variables used in FULL modeling, the number of variables was markedly reduced to 29, accounting for only 1.57% of the FULL. As shown in Figure 8, from the perspective of the distribution intervals of the selected variables, the feature wavelengths retained by MLLEISWS were mainly concentrated in several key spectral regions associated with protein content. This indicates that the method can markedly compress the number of wavelength variables while preserving effective information closely associated with the prediction of soybean protein content.

In this study, three classical wavelength selection methods, CARS, SPA, and UVE, were adopted for feature screening, and the screening results were comparatively analyzed with those obtained by the MLLEISWS method. The results showed that CARS, SPA, and UVE selected 49, 51, and 189 feature wavelength variables, respectively, as shown in Figure 8. Overall, MLLEISWS selected the fewest wavelength variables and thus demonstrated the strongest variable compression ability. By contrast, UVE retained a much larger number of wavelength variables. Figure 8 illustrates the distribution of feature wavelengths selected by different methods over the FULL range and their correspondence to the spectral profile. Although the sparsity of the selected variables differed among algorithms, the selected variables clustered in several common wavenumber regions, suggesting that these regions may contain key information related to protein content. Specifically, the wavelength variables selected by the four methods were mainly concentrated in five regions: 11,484.57–10,644.24 cm^−1^, 8885.30–8160.31 cm^−1^, 7015.14–6545.55 cm^−1^, 6133.62–5647.54 cm^−1^, and 4543.57–4086.33 cm^−1^. These regions are usually associated with overtone and combination bands absorptions caused by protein-related chemical bond vibrations. Among them, 11,484.57–10,644.24 cm^−1^ and 8885.30–8160.31 cm^−1^ correspond to the third and second overtone regions of -CH, -CH_2_, and -CH_3_ groups, respectively. The region 7015.14–6545.55 cm^−1^ is mainly related to the second overtone region of nitrogen-containing groups such as -CONH_2_ and -NH_2_. The range 6133.62–5647.54 cm^−1^ corresponds to the first overtone region of -CH, -CH_2_, and -CH_3_ groups. The range 4543.57–4086.33 cm^−1^ mainly corresponds to the combination bands of -CH, -CH_2_, and -CH_3_ groups. Overall, the feature wavelengths selected by different algorithms were concentrated in overtone and combination regions related to protein structure, indicating that the selected variables had a reliable spectral basis and provided key variable subsets for subsequent model construction.

### 3.3. Model Interpretability Analysis

**Figure 9:**
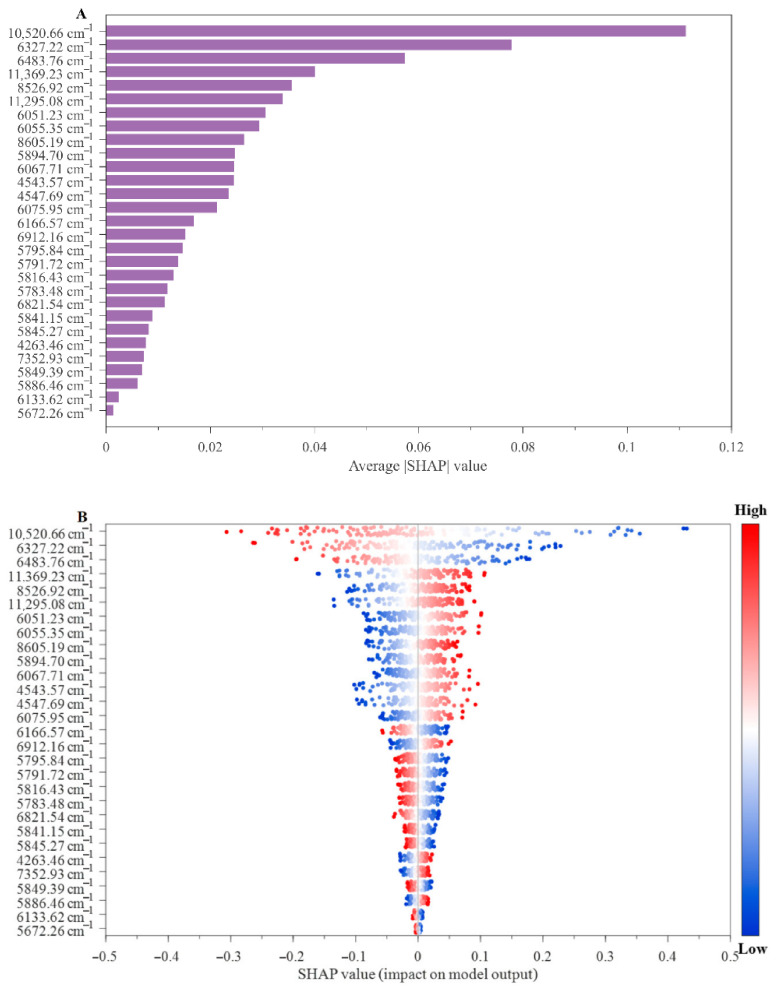
SHAP analysis of the PLS model based on the multi-linear ensemble wavelength selection method: (**A**) mean SHAP values showing feature importance, and (**B**) SHAP value distribution illustrating feature effects and their relationships with feature values.

To clarify how the feature wavelengths selected by MLLEISWS affected soybean protein content prediction, SHAP was used for interpretability analysis of the selected features. As shown in Figure 9A, the mean contributions of different feature wavelengths to the model output varied considerably. The ten wavenumbers with the highest mean absolute SHAP values were mainly 10,520.66 cm^−1^, 6327.22 cm^−1^, 6483.76 cm^−1^, 11,369.23 cm^−1^, 8526.92 cm^−1^, 11,295.08 cm^−1^, 6051.23 cm^−1^, 6055.35 cm^−1^, 8605.19 cm^−1^, and 5894.70 cm^−1^. Combined with the feature wavelength distribution and the corresponding absorption regions shown in Figure 8, these wavenumbers were mainly located in the first, second, and third overtone regions related to protein, indicating clear spectrochemical significance. Among them, 10,520.66 cm^−1^, 11,369.23 cm^−1^, and 11,295.08 cm^−1^ were mainly distributed near the region 11,484.57–10,644.24 cm^−1^, corresponding to the third overtone region of -CH, -CH_2_, and -CH_3_ groups. The bands at 8605.19 cm^−1^ and 8526.92 cm^−1^ were located near 8885.30–8160.31 cm^−1^ and mainly corresponded to the second overtone region of -CH, -CH_2_, and -CH_3_ groups. The wavenumbers 6483.76 cm^−1^ and 6327.22 cm^−1^ were located near 7015.14–6545.55 cm^−1^ and corresponded to the second overtone region of -NH_2_ and -CONH, containing nitrogen groups, which are closely related to amide bonds and amino structures in proteins. The bands at 6055.35 cm^−1^, 6051.23 cm^−1^ and 5894.70 cm^−1^ cm^−1^ were distributed near 6133.62–5647.54 cm^−1^ and corresponded to the first overtone region of -CH, -CH_2_ and -CH_3_ groups. Overall, the feature wavelengths ranking among the top in SHAP importance rankings were mainly distributed in the characteristic absorption regions related to protein-associated groups, indicating that the feature selection results of MLLEISWS had sound spectrochemical meaning and provided an interpretive basis for the model prediction results.

The SHAP beeswarm plot in Figure 9B further illustrates the directional influence of individual feature wavelengths on the model output. In this plot, the horizontal axis represents the SHAP value. SHAP > 0 indicates that the wavelength variable pushes the model prediction upward for that sample. SHAP < 0 indicates that the variable pushes the prediction downward. The point color changes from blue to red, indicating that the spectral response intensity at that wavelength increases from low to high [43]. The results indicated that different feature wavelengths could have either positive facilitating effects or negative suppressive effects on the model output. Specifically, higher response values at 10,520.66 cm^−1^, 6327.22 cm^−1^, and 6483.76 cm^−1^ were more frequently associated with negative SHAP values, indicating that high responses at these bands tended to contribute to lower predicted protein content. In contrast, higher response values at 11,369.23 cm^−1^, 8526.92 cm^−1^, and 11,295.08 cm^−1^ were more frequently associated with positive SHAP values, suggesting that high responses at these bands tended to contribute to higher predicted protein content.

From the perspective of group assignment and wavelength distribution characteristics, 11,369.23 cm^−1^, 11,295.08 cm^−1^, and 8526.92 cm^−1^ were mainly located in the third or second overtone absorption regions of -CH, -CH_2_, and -CH_3_ groups. Changes in spectral response in these regions more readily corresponded to the characteristic information of high-protein samples in the current sample system and therefore showed positive contributions. By contrast, 10,520.66 cm^−1^ was located near the third overtone region of -CH, -CH_2_, and -CH_3_ groups, while 6327.22 cm^−1^ and 6483.76 cm^−1^ were close to the second overtone region of nitrogen-containing groups. As these regions are more likely to be jointly affected by absorption band overlap and sample matrix differences, their higher response values in the current model more readily corresponded to the characteristic information of low protein samples, and therefore showed negative contributions.

Overall, the SHAP analysis not only revealed the importance ranking of the key feature wavelengths but also clarified the positive and negative directions of their contributions to protein content prediction. These highly contributive wavelengths were mainly concentrated in overtone and combination bands regions associated with the protein backbone and related functional group vibrations, which further strengthens the validity and interpretability of the feature wavelengths selected by MLLEISWS.

### 3.4. Modeling Results and Analysis

To compare the modeling performance of different wavelength selection strategies, PLS models were built using the feature wavelengths selected by CARS, SPA, UVE, and MLLEISWS, respectively, and compared with the FULL model. $R^{2}$, RMSE, rRMSE, and RPD were used as evaluation indices. The modeling performance of different methods is shown in Table 3.

As shown in Table 3, the CARS algorithm selected 49 wavelengths, accounting for 2.66% of the 1845 FULL variables. SPA selected 51 wavelengths, accounting for 2.76% of the FULL. UVE selected 189 wavelengths, accounting for 10.24% of the FULL. The MLLEISWS algorithm selected 29 wavelengths, accounting for 1.57% of the FULL. All four algorithms achieved dimensionality reduction to different extents, and MLLEISWS obtained the best overall performance while using the fewest wavelength variables.

The PLS models based on different wavelength selection methods showed clear differences in performance for soybean protein content prediction. The FULL model contained all 1845 wavelength variables and exhibited relatively weak predictive performance. In the training set, $R_{c}^{2}$ was 0.909, RMSEC was 0.584%, and rRMSEC was 1.56%. In the test set, $R_{p}^{2}$ was 0.891, RMSEP was 0.646%, rRMSEP was 1.72%, and RPD was 3.075. These results indicate that the FULL variables contained substantial redundant information and collinear variables. Although the FULL can represent the overall spectral characteristics of the samples, it also weakens model robustness and generalization to some extent.

By contrast, CARS improved predictive performance while retaining only 49 wavelength variables. $R_{c}^{2}$ was 0.942, $R_{p}^{2}$ was 0.914, RMSEC was 0.489%, RMSEP was 0.583%, rRMSEC was 1.32%, rRMSEP was 1.55%, and RPD was 3.404. These results indicate that the competitive elimination mechanism of CARS, which is based on regression-coefficient weighting, can preferentially retain dominant information more closely associated with the response variable [44]. However, as NIR absorption bands are broad and adjacent wavelengths are highly correlated, CARS may still retain groups of correlated variables around absorption bands, leaving a certain degree of redundancy.

SPA selected 51 wavelength variables. In the training set, $R_{c}^{2}$ was 0.929, RMSEC was 0.540%, and rRMSEC was 1.45%. In the test set, $R_{p}^{2}$ was 0.920, RMSEP was 0.561%, rRMSEP was 1.50%, and RPD was 3.538. These results indicate that when spectral information is distributed across multiple regions and variable correlation is strong, SPA selects wavelength variables based on a decollinearity criterion. Although it can alleviate collinearity, the selected variables tend to emphasize differences among spectral regions and may not simultaneously preserve protein-related informative signals as fully as possible. Therefore, some repetitive or redundant information may still remain among the selected wavelengths [45].

Compared with the other methods, UVE selected 189 wavelength variables, much more than CARS and SPA. Its model yielded $R_{c}^{2}$ of 0.923, $R_{p}^{2}$ of 0.917, RMSEC of 0.560%, RMSEP of 0.571%, rRMSEC of 1.51%, rRMSEP of 1.52%, and RPD of 3.476. Although its predictive ability was also better than that of the FULL model, the degree of model simplification was lower than that of CARS and SPA because more variables were retained, and its overall performance was slightly inferior to SPA. These results suggest that when spectral variables are highly correlated, UVE tends to retain more wavelength points judged by the algorithm to have stable contributions, some of which may contain repetitive information, thereby increasing the number of retained wavelengths [46].

In contrast, MLLEISWS selected only 29 feature wavelengths, yielding the smallest number of variables and the highest accuracy in the constructed regression model. In the training set, $R_{c}^{2}$ was 0.941, RMSEC was 0.490%, and rRMSEC was 1.32%. In the test set, $R_{p}^{2}$ was 0.933, RMSEP was 0.514%, rRMSEP was 1.37%, and RPD reached 3.863, indicating strong predictive ability and stability. This advantage mainly arises from the complementarity of the scoring criteria used by multiple linear learners. LASSO induces $L_{1}$ coefficient sparsity through regularization and can retain a small number of representative wavelength variables within strongly collinear spectral bands [47,48]. FSR uses a residual-correlation-driven stepwise updating mechanism and preferentially updates wavelength variables that show high absolute correlation with the current residual during iteration, so variables selected frequently tend to receive higher weights in the importance score [49]. Ridge regression adopts $L_{2}$ regularization to maintain stable coefficient estimation under collinearity and therefore tends to preserve the overall information within absorption-band regions [50,51]. PLS-based scoring measures wavelength importance based on the weights of individual wavelengths on the LVs and the explanatory contribution of each LV to the response variable, thereby reflecting the overall contribution of wavelengths to the response variable through the LV projection structure while accounting for both information integration in strongly correlated bands and modeling robustness [52]. Meanwhile, Huber provides a contribution measure from the perspective of marginal effects that is less sensitive to outliers and noise disturbance, thus providing a more robust supplement of marginal information for feature importance assessment [53,54].

MLLEISWS sums the wavelength importance scores from five linear learners. In essence, this is equivalent to an ensemble learning strategy for variable importance and consistency under multiple models and criteria, which reduces selection bias introduced by any single criterion and improves the stability and reproducibility of the screening results. In addition, this strategy constructs the initial variable set using the highest-ranked wavelengths in the integrated score, then gradually introduces subsequent candidate wavelengths according to their importance ranking. Combined with 10-fold cross-validation, the number of PLS principal components is re-searched, and the minimum RMSECV is used as the criterion to dynamically determine whether each newly added wavelength should be retained. This procedure not only preferentially preserves key information that contributes strongly to the target variable, but also effectively prevents redundant variables and interference wavelengths from entering the model. As a result, it enables efficient simplification of the feature subset, improves the stability of wavelength selection, improves model parsimony, and strengthens model generalization ability.

**Figure 10:**
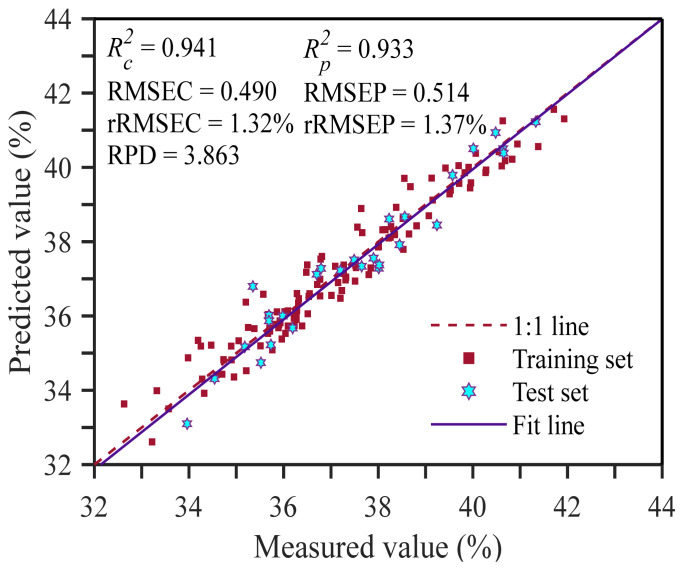
Scatter plot for model performance evaluation.

Figure 10 shows the scatter plots of predicted versus measured values for the MLLEISWS model in the training and test sets. The sample points from both sets are generally distributed close to the 1:1 line, and the fitted line is highly consistent with the 1:1 line, with no obvious systematic deviation, indicating good regression performance and stability. To further examine whether there was systematic bias in the test set, the MATLAB *t*-test function was used to test the difference between predicted and measured values in the test set. The test result was 0, and the significance probability *p* value was 0.438. The test statistic was smaller than the critical value, P_0.05_(27) = 2.052. These results indicate that the difference between predicted and measured values in the test set was not significant, suggesting that the model did not exhibit significant bias in the test set and that the overall regression accuracy was high.

### 3.5. Advantages and Limitations

Compared with conventional chemical assays, NIRS enables rapid and nondestructive evaluation of protein content and is suitable for large-scale sample screening and quality grading. However, in practical modeling, FULL variables are numerous and highly correlated across spectral regions, which can easily introduce redundant information and thereby impair model generalization [55]. To address this issue, this study combined MLLEISWS with PLS modeling and compressed the high-dimensional FULL variables into a small number of representative feature wavelengths, thereby reducing variable redundancy and collinearity while improving predictive performance. As the variable scale was markedly reduced, the corresponding computational and deployment costs were also lowered, making the method more suitable for prediction and system integration on resource-constrained hardware platforms and providing a feasible algorithmic route for subsequent embedded deployment and online detection.

From the perspective of the feature selection mechanism, wavelength selection methods such as CARS, SPA, and UVE usually rely on a single screening criterion, and under strongly correlated bands and noise disturbance the selected wavelengths may still contain residual redundancy [18,36]. In contrast, MLLEISWS introduces multiple linear learners to evaluate wavelength importance from different criteria and integrates the normalized scores to form a comprehensive importance ranking based on multi-criterion consistency. In this way, it reduces the selection bias caused by a single criterion and improves both the stability of the screening results and their suitability for high-accuracy modeling.

Although the method demonstrated good application potential, the generalizability of the conclusions is still constrained by sample coverage and modeling assumptions. While the samples in this study covered multiple major soybean-producing regions of China and formed a certain protein-content gradient, the overall sample size was still limited, and the samples were mainly Chinese soybean materials. Samples from other countries or regions were not included. Therefore, the applicability of the model under different genetic backgrounds and ecological conditions still requires further verification. To address this limitation, future studies will include soybean samples from different countries, ecological regions, genetic backgrounds, and production systems to further evaluate the external generalization ability of the model. From the perspective of methodological assumptions, MLLEISWS and its five learners are mainly based on a linear scoring framework, and PLS is also a linear regression method. For scenarios involving nonlinear relationships, the ability of linear models to characterize complex mappings may be limited. As sample size increases and acquisition conditions become more complicated, nonlinear methods such as deep learning may offer potential advantages in feature representation. Future studies will expand the sample size across multiple regions and countries and further explore nonlinear modeling strategies to improve applicability under more complex conditions.

## 4. Conclusions

This study was aimed at the rapid and nondestructive determination of soybean protein content. Four wavelength selection methods, CARS, SPA, UVE, and MLLEISWS, were used to select feature wavelengths, and PLS quantitative models developed under different strategies were constructed and comparatively analyzed. An interpretability analysis of the key wavelengths selected by the MLLEISWS model was further conducted using SHAP. Among the NIR models developed for soybean protein content using different wavelength selection strategies, the PLS model established with MLLEISWS showed the best overall performance. Under the condition that only 29 feature wavelengths were retained, the model achieved an rRMSEP of less than 1.40% in the test set, demonstrating very high predictive accuracy and good stability while effectively reducing dimensionality. SHAP analysis indicated that the selected key wavelengths were mainly located in characteristic spectral regions associated with protein chemical composition, suggesting that model predictions were primarily driven by effective spectral information closely related to soybean protein content and therefore showed good chemical rationality and interpretability. While simplifying the feature variable set, MLLEISWS can effectively improve model predictive accuracy and provide good model interpretability. It can provide technical support for the rapid and nondestructive determination of soybean protein content and serve as a reference for the construction of NIR quantitative analysis models for agricultural product quality.

## Figures and Tables

**Table 1:** Results of different spectral preprocessing methods.

Preprocessing Methods	$R_{cv}^{2}$	RMSECV (%)	RPDCV	LV
RAW	0.895	0.599	3.503	10
SG	0.900	0.588	3.562	9
MSC	0.850	0.716	2.934	9
SNV	0.851	0.715	2.944	9
WD	0.901	0.582	3.613	10
FT	0.899	0.589	3.558	9
Deconv	0.898	0.588	3.558	9
MAS	0.899	0.585	3.568	9
SG+MSC	0.850	0.714	2.938	9
SG+SNV	0.852	0.711	2.953	9
SG+WD	0.900	0.583	3.586	10
SG+FT	0.899	0.588	3.557	9
SG+Deconv	0.899	0.588	3.552	9
SG+MAS	0.897	0.591	3.547	10
MSC+SNV	0.850	0.715	2.945	9
MSC+WD	0.855	0.701	2.999	9
MSC+FT	0.851	0.710	2.956	9
MSC+Deconv	0.851	0.711	2.951	9
MSC+MAS	0.850	0.714	2.938	9
SNV+WD	0.856	0.699	3.007	9
SNV+FT	0.852	0.708	2.966	9
SNV+Deconv	0.851	0.711	2.947	9
SNV+MAS	0.850	0.712	2.948	9
WD+FT	0.900	0.582	3.590	10
WD+Deconv	0.899	0.585	3.565	9
WD+MAS	0.900	0.584	3.577	10
FT+Deconv	0.897	0.592	3.534	10
FT+MAS	0.899	0.587	3.577	9
Deconv+MAS	0.899	0.588	3.550	9

$R_{cv}^{2}$ and RPDCV are the cross-validation $R^{2}$ and RPD, respectively.

**Table 2:** Sample set information.

Sample Set	Number	Min (%)	Max (%)	Mean (%)	Standard Deviation (%)	Variation Coefficient (%)
All samples	143	32.63	41.93	37.233	2.027	5.40%
Training set	115	32.63	41.93	37.161	2.031	5.50%
Test set	28	33.96	41.33	37.527	2.022	5.40%

**Table 3:** PLS regression models for soybean protein content.

Wavelength Selection Method	FULL	CARS	SPA	UVE	MLLEISWS
Number of wavelengths	1845	49	51	189	29
$R_{c}^{2}$	0.909	0.942	0.929	0.923	0.941
$R_{p}^{2}$	0.891	0.914	0.920	0.917	0.933
RMSEC (%)	0.584	0.489	0.540	0.560	0.490
RMSEP (%)	0.646	0.583	0.561	0.571	0.514
rRMSEC (%)	1.56	1.32	1.45	1.51	1.32
rRMSEP (%)	1.72	1.55	1.50	1.52	1.37
RPD	3.075	3.404	3.538	3.476	3.863
LV	8	8	9	9	11

$R_{c}^{2}$, $R_{p}^{2}$, RMSEC, RMSEP, rRMSEC, and rRMSEP represent the $R^{2}$, RMSE, and rRMSE for the training and test sets, respectively.

## Data Availability

The original contributions presented in this study are included in the article. Further inquiries can be directed to the corresponding author.

## Abbreviations

The following abbreviations are used in this manuscript:

CARS	Competitive adaptive reweighted sampling
Deconv	Spectral deconvolution
FSR	Forward stagewise regression
FT	Fourier transform
FULL	Full-spectrum
Huber	Huber regression
LASSO	Least absolute shrinkage and selection operator
LV	Latent variable
MAS	Moving average smoothing
MCCV	Monte Carlo Cross-Validation
MLLEISWS	Multiple linear learner ensemble importance-score wavelength selection
MSC	Multivariate scattering correction
NIRS	Near-infrared spectroscopy
PLS	Partial least squares
PRESS	Predicted residual error sum of squares
RAW	Raw spectra
R^2^	Coefficient of determination
Ridge	Ridge regression
RMSE	Root mean square error
RMSECV	Root mean square error of cross-validation
RPD	Residual predictive deviation
RPDCV	Residual predictive deviation of cross-validation
rRMSE	Relative root mean square error
SG	Savitzky–Golay smooth
SHAP	Shapley additive exPlanations
SNV	Standard normal variate
SPA	Successive projections algorithm
UVE	Uninformative variable elimination
VIP	Variable importance in projection
WD	Wavelet denoising
